# Determinants of physical activity promotion in primary care from the patient perspective of people at risk of or living with chronic disease: a COM-B analysis

**DOI:** 10.1186/s12875-024-02440-2

**Published:** 2024-05-28

**Authors:** Aisling McGrath, Barry Lambe, Evan Matthews, Karolyn McDonnell, Michael Harrison, Bróna Kehoe

**Affiliations:** 1 Centre for Health Behaviour Research, Department of Sport and Exercise Science, South East Technological University, Waterford, Ireland; 2 National Centre for Men’s Health, South East Technological University, Carlow, Ireland

**Keywords:** PA promotion, Healthcare, Chronic disease, Behaviour Change, COM-B model

## Abstract

**Background:**

Chronic disease (CD) accounts for more than half of the overall global disease burden and physical activity (PA) is an established evidence-based strategy for the prevention and management of CD. Global policy emphasises the value of embedding PA into primary healthcare, highlighting the positive effects on PA behaviour. However, there is limited implementation of PA protocols in primary care, and research is needed to guide its integration into routine practice. The voice of the patient is underrepresented in the literature, resulting in the absence of critical insights into determinants of PA promotion in primary care. The purpose of the research was to identify the perspectives of people at risk of or living with CD on the determinants of PA promotion in primary care and to map these determinants across the six COM-B constructs.

**Methods:**

Semi-structured interviews (*n* = 22), guided by the COM-B model were conducted with people aged 35–60 years, at risk of or living with CD and not meeting the PA guidelines. A hybrid analytic approach of thematic inductive and deductive analysis was applied to the participant transcripts guided by a COM-B informed coding framework.

**Results:**

In total, 37 determinants across constructs related to capability, opportunity and motivation were prominent, examples include; physical capability constraints, the conflation of exercise with weight management, credibility of the health services in PA advice, communication styles in PA promotion, expectations of tailored support for PA, social support, accessibility, and integration of PA into routine habits.

**Conclusion:**

Exploring the determinants of PA promotion through the lens of the COM-B model facilitated a systematic approach to understanding the primary care user perspective of the healthcare professional (HCP) broaching the topic of PA. Findings emphasise the value of HCPs being supported to broach the issue of PA in a therapeutic and patient-centred manner using diverse and flexible approaches, while highlighting the importance of tailored, accessible PA opportunities that build self-efficacy and foster social support. The research provides valuable learnings to support PA promotion and the development of strategies in primary care through encompassing the perspective of those living with or at risk of CD.

**Supplementary Information:**

The online version contains supplementary material available at 10.1186/s12875-024-02440-2.

## Background

Chronic disease (CD) are long term medical conditions that require continuous medical attention and/or limit activities of daily living. CD accounts for greater than half of the overall global disease burden [1]. With the growing ageing population, this burden is expected to continue to rise [2]. One of the primary risk factors for the development of CD is physical inactivity [3]. Globally, 27.5% of adults do not meet the recommended level of physical activity to maintain health [4]. This figure is substantially higher in high-income countries, in older adult populations and in those living with CD [5, 6]. Physical inactivity is estimated to be responsible for between 6 and 10% of the major CDs globally [7].

Physical activity (PA) is a powerful tool for the primary and secondary prevention of CD [8]. Engaging in the recommended level of PA is associated with a decrease in the risk of all-cause mortality by approximately 30% [9]. Exercise, a structured form of PA, can have a positive effect on clinical outcomes, physical functioning, psychological wellbeing and quality of life in a wide range of CDs [10]. The role of PA in the prevention and control of CD is recognised internationally, including by the World Health Organisation (WHO) [11] and the Centres for Disease Control and Prevention [12]. Healthcare professionals (HCPs) in primary settings are considered desired, credible and well-placed messengers for the delivery of PA advice, brief intervention and referral and have the capacity to play an integral role in the promotion of PA behaviours among their patients [13]. Primary care users are interested in discussing PA with their HCP [14] and promotion of PA in a primary care setting can have positive effects on PA behaviour [15]. Indeed, integrating PA and CD prevention into primary health care systems is one of the best investments for PA [16]. In the Global Action Plan on Physical Activity, implementing and strengthening systems to increase PA in healthcare sectors is one of twenty policy actions proposed [11].

Despite this evidence and international support, only 40% (approximately) of countries report having national primary healthcare protocols for the management of PA, and in one third of those countries, the protocols are used in less than half of health facilities [11]. In Ireland, Make Every Contact Count (MECC) is the national health behaviour change framework for HCPs, which sets how brief advice and brief intervention in relation to physical activity, smoking, alcohol and diet behaviours should become part of routine clinical care [17]. Integrating PA into healthcare systems requires investigation of the multiple determinants of successful implementation of PA counselling and subsequent referral/signposting pathways across an ecological model of implementation. Primary care providers have cited lack of time, incentives and PA knowledge; belief’s that patients wouldn’t engage with the advice and medico-legal concerns around referral to community services as barriers to PA promotion [18]. Primary care users have described being open to their HCP raising the topic of PA but disliking being dictated to [19]. They express a sense of legitimacy in being referred to PA by the HCP and favour being connected to specific opportunities. Individually tailored advice is considered more acceptable, both in terms of the sensitivity of its delivery and its appropriateness [20]. However, the voice of the primary care users is less well represented in the literature compared to that of HCPs, in particular those living with CD [21].

In order to determine an acceptable model for integrating PA into healthcare systems, it is important to include the voice of those whom the initiative is ultimately meant to benefit, as this is a critical determinant of acceptability and ultimate success [22, 23]. It is well established that CD is a result of complex determinants of health and relatedly, those who are at risk of or living with CD face multiple complex barriers in modifying lifestyle behaviours [24]. In order to maximise engagement of those at risk of or living with CD with PA opportunities, it is important to investigate the factors that influence their PA behaviour and how these factors can be moderated within the primary health care setting.

The use of a suitable framework that offers a systematic way to understand PA behaviour in this context can assist in identifying an acceptable approach to integrating PA into primary healthcare systems.

The COM-B is a model that encompasses behaviour change theory and can be utilised to identify what is needed for a behaviour change intervention to be effective as well as to determine if an intervention is effective [25]. The COM-B system considers the capability, opportunity and motivation of individuals as essential catalysts that can enable or inhibit behaviour change. It operates within a behaviour change wheel (BCW) that considers intervention functions aimed at addressing deficits in capability, opportunity and motivation, as well as an outer wheel that considers policy level determinants that can impact interventions and subsequent behaviour. The model has been recognised as an effective tool that can accurately capture important determinants across multiple levels of implementation and is recognised as a valid approach in terms of the development of behaviour change theory and implementation science [26, 27].

The COM-B model was, therefore, used to guide the research design for this qualitative study of PA in the primary care setting. More specifically, the aim of this research was to identify perspectives of people at risk of living or living with CD on the determinants (i.e. barriers or facilitators) of PA promotion in primary care and to map these determinants across the six COM-B constructs.

## Methods

### Research design and context

This research was conducted as part of a broader project commissioned by the national health service in Ireland, the Health Service Executive (HSE), that sought to inform the development of standardised pathway for PA in primary care settings in Ireland. Prior to the research commencement, extensive consultation was carried out with key representatives from the HSE with a remit for policy and practice development on PA promotion in Ireland. This collaborative process allowed for the refinement of the research question(s), design and approach within the current study.

This research employed a descriptive qualitative approach [28, 29], with an inquiry that was framed using the COM-B framework [25, 29]. Notwithstanding this consultation process, the research team had independent responsibility and autonomy for data analysis and reporting.

### Sampling and recruitment

This study used a combined voluntary response and quota sampling approach during recruitment. In this way, the research sought to recruit eligible volunteers that gave balanced representation to gender, geographical residence and nature of residence (urban or rural) in the Republic of Ireland. The HSE acted as gatekeeper for a recruitment advertisement process. Using official health service social media accounts, key recruitment and participation information was disseminated online between March to May 2023. Study information was also distributed to coordinators of specific CD management support and education programmes and to specific national health service forums with a remit for gender specific health promotion to support gender balance during recruitment. The dissemination material included a link to a short participant eligibility form, which captured key demographic inclusionary criteria (age, gender, PA levels and whether they were resident in the Republic of Ireland). Interested persons were asked to provide their contact details and to give consent for a member of the research team to contact them for a call-back. Eligible participants received information and an informed consent form via email. In addition to quota sampling techniques applied, further inclusion criteria were, people aged 35–60 years, living with CD (defined as a clinical condition that lasts ≥ 1 year and requires ongoing medical attention or limits activities of daily living or both) *and* not meeting the PA guidelines according to the WHO [28]); *or* at risk of CD (defined as not meeting the PA guidelines according to the WHO [28]); and living in the Republic of Ireland. Research ethics approval for this study was provided by the South East Technological University’s School of Health Sciences Waterford Research Ethics Committee.

### Data collection

Following consent, the research team undertook online semi-structured interviews using Zoom video communications platform and were video and audio-recorded. Initially upon interview commencement, demographic and health information regarding the nature of CD (by displaying a showcard, from the Healthy Ireland Interview Instrument [30]) were gathered.

To address the primary aim, an interview topic guide (Supplementary File 1) was developed by the authors for the purpose of this study using the COM-B model and open-ended questions allowed for other concepts beyond these constructs to be explored. In using the COM-B model, the research focused on participants with or at risk of CD in relation to their physical and psychological capability, their reflective and automatic processes of motivation, as well individual opinions on the social and physical opportunities available in relation to their experience of PA promotion in the primary health care setting. In this way, participants were asked about interactions with key community-based HCP (General Practitioner (GP), practice nurse, occupational therapist or dietician) and their preferences for these interactions. At the outset of the interview, PA was defined as interchangeable with exercise and examples of PA were provided (e.g. walking for transport or recreation, gardening, cycling, yoga, swimming, jogging and running).

Interviews were 60–75 min in duration. Interviews were conducted by KM, who is trained and experienced in qualitative research. Sample size was determined by data saturation, the point at which no new information was gained.

### Data analysis

The interviews were transcribed by KM and a coding framework based on COM-B constructs, techniques cited in the behaviour change taxonomy, definitions and guidelines was developed by AMc to guide consistent coding of qualitative data. A hybrid analytic approach of thematic inductive and deductive analysis was applied [31], aligning with the qualitative descriptive approach outlined earlier. This meant that whilst the COM-B model was applied to inform outcomes, the analysis process remained open to findings outside of those pre-set domains. Initially, three members of the research team (AMc, BL, EM) used the coding framework to independently code three separate transcripts. This was followed by a calibration exercise to resolve coding discrepancies and to foster inter-rater reliability. The remainder of the transcripts were coded independently by the AMc, BL, EM with frequent communication between researchers to refine the coding framework and ensure consistency in its application.

Following this extensive coding process, the research team (AMc, BL, EM, BK) undertook a collaborative mapping exercise to define and label determinants, and further map these determinants to the 6 components of the COM-B model. Determinants refer to the factors or influences that shape an individual’s behaviour regarding PA promotion within the primary care setting. The results were presented through the frame of the COM-B model as it provides a systematic approach to examining the factors that influence behaviour and behaviour change. Structuring the results in this fashion facilitates a representation of the interplay between capability (e.g. participants’ awareness of exercise as medicine for CD), opportunity (access to PA resources and facilities) and motivation (e.g. personal beliefs and attitudes towards PA). In this way, subsequent interventional efforts must consider determinant level interactions. The findings presented provide a comprehensive understanding of the experience of participants as well as their needs in relation to PA support in primary care settings in Ireland.

## Results

A total of 22 interviews were completed and participants had a mean age of 49.18 ± 5.83 years, ranging from 40 to 60 years. There was an even representation of males and females and of those living in an urban and rural setting. Almost all participants had follow-on education beyond post-primary, with the majority having degree or postgraduate qualifications. The majority (*n* = 16; 72.7%) of participants reported at least one CD, primarily obesity (*n* = 7; 32%). All participants reported that they were not currently meeting the PA guidelines according to the WHO [28]. Participants discussed their current experience of being inactive and also their past experiences of being active and attempting to maintain PA. Participant characteristics are outlined in supplementary file 2. All participants reported that they were not currently meeting the PA guidelines according to the WHO [32]. Participants discussed their determinants of initiating PA currently but also past experiences of maintaining PA. In total, the analysis of data guided by the COM-B model, led to the deduction of 37 determinants across the respective constructs as. Determinants are highlighted in bold text and Table 1 provides a description of each determinant.

**Table 1:** Patient-informed determinants of engagement with PA promotion in primary care categorised by the COM-B model

Component	Sub-Component	Determinant	Description
Capability	*Physical capability*	Physical capability constraints	Physical ability or limitations that affect capacity for PA
	*Psychological Capability*	The perception of exercise as a form of medicine	Recognising the role of PA or exercise as a means to prevent, manage or treat health conditions
		The conflation of exercise with weight management	Perceiving exercise as synonymous with weight management, where the primary purpose of PA is believed to be controlling or altering body weight
		Recognising the value of movement beyond traditional exercise	Understanding the role any bodily movement can play in terms of contributing to PA outside of structured exercise
		Ability to understand and consume information about PA	The perception individuals had about their ability to understand and consume the different types of information being relayed about PA
		Demonstration of PA	Exercise practitioner demonstrating PA techniques to build self-efficacy
		Prompts and cues to be active	Receiving reminders and prompts to engage in PA
		Self-efficacy of individuals	Participants’ belief in their ability to be physically active
		Previous experience	Past interactions with PA and HCPs as a determinant of future engagement
Opportunity	*Social Opportunity*	Level of support received in HCP-Patient dynamic	Patient beliefs about HCPs and whether they focus on instructing patients without providing sufficient support to increase PA levels
		Credibility of the health services in PA advice	Perception of the credibility of health services encompassing trustworthiness of GPs, HCPs and the Health Service Executive as a credible source of information
		Communication styles in PA promotion	Preference of individuals for a collaborative approach eliciting the patient’s own motivations for PA versus a more directive style where HCPs inform patients about the reasons for increasing PA
		Balancing fear appeals in PA communication	Preference of individuals as to whether discussions with a HCP about the negative consequences of inactivity or the positive benefits of PA have a more compelling impact
		Therapeutic alliance as central to PA conversations	The importance of a collaborative and trusting relationship between a HCP and patient in initiating meaningful conversations about PA independent of communication style employed by HCP
		Active referral process for PA programmes	The necessity for a more involved and structured referral process to facilitate patient engagement with PA programmes versus passive methods
		Capacity constraints of GPs in PA support	Participants belief about the limited capacity of GPs to sufficiently support patients to modify their PA behaviour
		Expectations of tailored support for PA	The perceived important of tailoring the exercise programme to the individual’s medical condition and unique preferences
		Social support in PA programmes	The role of social support in promoting a sense of solidarity, motivation and belonging that facilitates initiation and maintenance of PA
		Vicarious experience in PA	Witnessing relatable individuals PA achievements, which fosters a sense of commonality and subsequent motivation
		Obligation to others in PA programmes	Individual’s sense of obligation to others in group PA which creates a psychological contract facilitating commitment to PA
		Financial commitment to PA	The role of paying to participate in PA in promoting a sense of accountability and commitment
	*Physical Opportunity*	Awareness of local PA opportunities	The significance of enhancing awareness of PA opportunities in the locality
		Structured and meaningful PA programmes	The necessity for structured and purposeful PA programmes beyond awareness in facilities and locations
		Accessibility of PA programmes	Accessibility of PA programmes in terms of location, literacy and cost
		The referrer as a safety mechanism	The role of the referrer as a conduit to PA for those with low self-efficacy
Motivation	*Reflective Motivation*	Integration of PA into routine habits	Integrating PA into routine habits
		Competing priorities	Prioritisation of PA in the face of a busy lifestyle, caring and professional commitments
		PA goal setting	Setting specific and achievable goals as to promote a sense of achievement about PA
		Self-monitoring of PA	Opportunities to monitor own progress towards PA goals
		Feedback on PA behaviour	Receiving feedback on PA performance or progress from a HCP, acknowledging achievements and offering advice or adjustments for improvement
		Incentivisation of PA	Material rewards or incentives as a means to motivate individuals to maintain PA
		Role of self-Compassion in PA engagement	Being kind and understanding toward oneself in moments of difficulty or failure in relation to PA
		Urgency for autonomy and self-awareness in health behaviour change	Participants’ beliefs that their current inactive lifestyle necessitated fundamental behaviour change
		Intention to be physically active	An individual’s conscious decision or readiness to perform PA within a specified timeframe
		Fear of morbidity and mortality	An emotional and cognitive perception of potential negative health outcomes associated with physical inactivity
	*Automatic motivation*	Self-consciousness in PA engagement	Concern individuals have about how they are perceived or judged while participating in PA
		Enjoyment of PA programmes	A subjective experience of pleasure, satisfaction, or positive affect associated with engaging in PA

### Capability

#### Physical capability

##### Physical capability constraints

Participants outlined participants’ physical abilities and limitations and factors such as cardio-respiratory fitness, strength, endurance, flexibility, and coordination significantly impacted their capability to engage in PA. These aspects were closely intertwined with their health conditions and disabilities:

*‘I’m no longer able to stand. So, if I need to be … like I used to go swimming years ago, but now you know, to go swimming is nearly out of the question’ AC,54 F*.

#### Psychological capability

##### The perception of exercise as a form of medicine

Recognising the role of PA or exercise as a means to prevent, manage or treat health conditions appeared to have an important role in terms of intention to be physically active. A minority of participants recognised the importance of PA for their health and demonstrated an awareness of the need to be physically active in terms of the potential benefits it could yield for their specific health conditions and the importance of making informed decisions in that regard:

*‘You have to take stock and look deeper into yourself and kind of say, you know, I need to do something here big time. Because this is catching on with me in next couple of years, not 20 or 30 years down the road, this could be visiting me sooner rather than later.’ TF, 41 M*.

##### The conflation of exercise with weight management

However, the majority of participants presented a limited narrative in terms of knowledge of how PA could support them with CD management. The conflation of exercise with weight management suggested limited awareness of other potential benefits related to their respective health conditions or disease prevention and management. Participants also highlighted the approach of the HCP in this regard, where in particular the GP’s perspective on exercise had an important role in shaping the participants beliefs around recognising its value:

*‘She [GP] would weigh me every time I would go in and then she’s like ‘You have to watch this now, you know, it’s creeping up. You have to get it back down’ AJ, 44F*.

##### Recognising the value of movement beyond traditional exercise

The majority of participants appeared to equate being active with traditional “exercise” and getting fit, whereas recognising the distinction between movement and exercise, particularly the importance of movement in the face of physical limitations appeared to influence the capability of participants to be physically active. This appeared to be further compounded by the fact that any discussion participants reported in relation to PA with HCPs was in relation to ‘exercise’ as opposed to ‘movement’:

*‘I’m kind of restricted in what I can do. Well cardio definitely, as far as going into a gym, bench press 100 kilos a day? I just had a heart attack. And under strict doctor’s order to do a lot of exercise, but no heavy lifting.’ PC, 49 M*.

##### Ability to understand and consume information about PA

The perception individuals had about their ability to understand and consume the different types of information being relayed about PA also impacted their perceived capability. Some participants expressed a need for ‘clarity of messaging’ to prevent them feeling overwhelmed:

*‘I find GPs, they have loads of leaflets on all sorts of conditions and stuff. And I just think I might just get lost.’ JD, 52 M*.

##### Self-efficacy of individuals

Many participants identified with having low self-efficacy or belief in their ability to initiate and sustain PA and this was a barrier to engagement with PA out of fear of failure, fear of the unknown or sense of shame and vulnerability:

*‘If it’s a walk I’m thinking ‘Oh God’, the first thing they’re going to do is hike up a high mountain. And I’m gonna be thinking like, there is always that fear of not fitting in, or maybe that what they do is maybe beyond my capacity at the moment, and maybe the fear of kind of making a show of myself for want of a better word.’ JD, 41 M*.

##### Demonstration of PA and; prompts and cues to be active

Indeed, participants’ perceptions of the level of skill needed to ‘exercise’ rather than ‘move’ impacted their self-efficacy further. Many participants also reflected on their need to build self-efficacy through the guidance of their HCP or other supports. Participants cited the importance of an exercise practitioner demonstrating PA techniques to build-self-efficacy as well the role of reminders and prompts to engage in PA:

‘*But I know, walking for 30 minutes is only going to help. So maybe it’s excuses. I think, well, if there’s a platform, maybe that will help and I’ll be tracked and but I need something. I’m having a little bit of a block in my mind.’ BL, 52 F*.

##### Previous experience

Relatedly, previous experience also played a prominent role in the participants perceived self-efficacy and their subsequent sense of psychological capability to be physically active. This encompassed both past interactions with their HCP and PA. Positive previous experiences with PA contributed to a sense of self-efficacy for participants where conversely, negative experiences created a deterrent to future PA engagement. Positive experiences with their HCP also enhanced the participants motivation to be physically active, particularly where helpful advice or guidance was offered. When participants felt respected by their HCP they felt a sense of empowerment:

*‘I could ask the same thing in a nice manner as well. And I would get “oh, yeah, yeah, we’ll come back to you on that” or whatever. It isn’t right that, you know, when I’m well able to speak up for myself. And just because I’m the patient, they won’t take me seriously, whereas here [community programme] they see me as a person that is very independent and well able to speak my mind and know what works and what won’t work.’ AC, 54 F*.

Negative experiences with their HCPs were incidences that left them feeling invalidated, shamed or judged, which disempowered them to be physically active.

*‘…he just basically put a measure tape around my stomach and said: “There’s your problem”, he said, and I found that really condescending.’ SD 49 M*.

Many participants reported having no previous experience of discussing PA with their HCP with participants commenting:

*‘When I think about its so gapingly absent, it’s quite phenomenal.’ CB, 49 F*.

*‘There was no follow up whatsoever. And I asked him, and he just said, ‘keep doing this, keep doing as much as you can’… I just happened to meet a good [physiotherapist] recently, but you know I was never scheduled into rehabilitation or anything. I was never told anything like that.’ JM, 41 M*.

### Opportunity

#### Social (interpersonal) opportunity

##### Level of support received in HCP-Patient dynamic

Social (interpersonal) opportunities are the result of external social factors such as cultural norms and social cues [22]. The level of support received in the HCP-Primary care user dynamic was an important determinant. Participants welcomed HCP-initiated discussions on PA but expressed dissatisfaction with a predominant *‘instructing without supporting’* approach. This approach was perceived as oversimplified and neglectful of the complexities involved in behaviour change. Many participants felt there was an overestimation of their PA knowledge, resulting in generic instructions lacking personalised support and suggested a need for more nuanced guidance:

*‘It’s not helpful, it’s not targeted. Like eat more fruit, eat more vegetables, do more exercise. You don’t need that, that’s not a prescription if you like. What might they have done differently? What might have been more supportive for you? And what’s available to support it. You know what I mean? Like, somebody says, like, just walk for half an hour a day. When you walk, what do you do when you’re walking? You know? Are you just ambling or do you need to keep a certain pace? Or, you know, what are the best apps on your phone or whatever it might be? And go over stuff like that, how to set a goal because, you know.’ AB, 53 M*.

##### Credibility of the health services in PA advice

However, participants emphasised the credibility of healthcare services, particularly GPs and people working within the HSE, the HSE-endorsed programmes, in providing trustworthy advice on PA. This sense of credibility meant that participants placed considerable weight on the advice to be active provided by their GP in particular. Being referred to a HSE-endorsed programme specifically for exercise served as a mechanism to filter out ‘*misinformation’* and the array of poor quality sources of information on PA:

*‘you know yourself, you’ve gone to Google and one [exercise programme/ trend] would criticize the other one will say, this one’s good. And you’ll say, that’s good. Yeah, it’s unreliable information. You don’t know what information is reliable. You know? Because, you know, they’re even saying that thing, thirty minutes now is not right. And some people say, No, it’s 20 minutes. And some people say you don’t exercise at all.’ GK, 58 M*.

##### Communication styles in PA promotion and; balancing fear appeals in PA communication

Balancing fear appeals in PA communication was an important determinant where there were polarised preferences of individuals as to whether discussions with a HCP about the negative consequences of inactivity or the positive benefits of PA have a more compelling impact:

*‘There’s no point in telling me well, if you don’t exercise, you might have a heart attack. Or if you don’t exercise, you might have a stroke. You know, if you said: “If you exercise or the likelihood of you having a stroke or whatever it is, is reduced by a factor of whatever it might [would appeal to me]’ AB, 53 M*.

##### Therapeutic alliance as central to PA conversations

Irrespective of the HCP communication style, the majority of participants cited the therapeutic alliance as being the most important determinant of a positive conversation about PA. A strong therapeutic alliance makes it easy and acceptable for the HCP to broach the topic of exercise. For the same reason, several participants thought it was acceptable for the HCP to transition to a more direct informing communication style once it was preceded by a brief, more collaborative approach:

*‘I would prefer to be told personally, to be told the information. Yeah. Because if I understand you correctly, the context you’re in, you’re only with a GP or a healthcare professional to get help. I don’t want to have a conversation. I don’t need to have a conversation with him. I mean, for whatever, that half an hour and no, I just need to be told what to do to get better. And that’s it.’ JM, 41 M*.

Conversely, others recognised the need to have a more in-depth conversation after PA is broached in the consultation but notably stressed that this conversation does not necessarily need to be with their GP:

*‘It needs to be a full-on conversation. Because then if you’re more engaged, you’re more willing to buy into the program. …You know the best salespeople will get you to buy anything, but they’ll engage you first before you sell it to you.’ TF, 41 M*.

##### Capacity constraints of GPs in PA support

The capacity constraints of GPs to support PA engagement was also acknowledged by participants as an important determinant. While many believed that this provides a strong rationale for creating a mechanism for signposting, it was also suggested that other HCPs, support staff and even digital components could play a role in starting the primary care user on the pathway:

*‘And his answer would just be, you know, come in, treat me, and, you know, that would be it - out the door. And he would just treat you for your ailments. They’re too busy.’ JC, 59 F*.

##### Active referral process for PA programmes

In relation to a referral process, participants were unanimous in their preference for active signposting. This includes the GP receiving feedback on their progress, thereby ensuring continuity of care. Passive signposting using posters or leaflets were thought of as unlikely to be effective, particularly where individual had responsibility to contact a PA programme:

*‘I know that if I’ve been to the doctor, and they’ve given me a form or something to look at, it’s going in my bag, I’m going to go back to work, I’m going to look at it maybe tonight or maybe on the weekend.’ BL, 52 F*.

##### Expectations of tailored support for PA

Participants emphasised the importance of individually tailored support for PA. There was an expectation that opportunities take into consideration individual health conditions and status and preferences:

*‘If I have this inclination that something is drawn up or made specifically for me, it becomes about me. So I tend to be like, ‘Okay, now I’m the center of attraction.’ Yes. So, for me that gives me that zeal to really want to, I want to put my name or my stamp on.’ OI, 50 F*.

This was predominantly related to participants’ perceived sense of safety and appropriateness of exercise programmes. Exercise programmes specifically designed for people with CD were desirable:

*‘There would be an expert that would have a look over it, and maybe take into account your own personal situation, like the risk of diabetes. I think that’s important. And it’s not just a general class and everybody that’s referred to same class. That there is some input from so many health care professionals into what would suit you personally. That’s really important.’ AJ, 44 F‘*.

##### Social support in PA programmes and; vicarious experience in PA

Participants described social support and vicarious experience as important determinants of both initiating and maintaining PA, where a sense of commonality creates solidarity, motivation and a sense of belonging. The aspects of relatability most frequently mentioned were age, gender, physical ability and health condition:

*‘I suppose the social aspect, I think is important …. because I do think it’s always harder on your own to do stuff. And if there was any people, particularly with chronic diseases, I could imagine if there’s a social aspect where you’re bringing together maybe ten people with a common issue, there is a kind of solidarity and support and people know what you can and cannot do, you know what I mean?’ JD, 52 M*.

##### Obligation to others in PA programmes and; financial commitment to PA

The creation of social support through group classes would help to create a psychological contract (expectation bordering on obligation) to be more active and instil a sense of accountability. The majority of participants were willing to pay a fee for a programme, which in turn would enhance their sense of accountability:

*‘If it’s a pre-recorded thing, I’m just not gonna go there. So, if it’s live, I generally have paid to go and so I’ll turn up to that.’ CB, 49 F*.

#### Physical (environmental) opportunity

##### Awareness of local PA opportunities and; structured a meaningful PA programmes

Physical opportunities are the result of environmental factors such as location, facilities, accessibility and resources [22]. There was a consensus that increasing awareness of people at risk of or living with CD of PA opportunities in their locality would be worthwhile. However, if these opportunities were limited to information about facilities and locations, this would not suffice to change behaviour. The PA opportunity would need to be a meaningful and structured programme:

*‘I’m not happy about that. Because I have something I need to work with mentally, for some reason. I have all the parks around me; I live in a lovely area. When I get home from work, I am just not wanting to do anything.’ BL, 52 F*.

##### Accessibility of PA programmes

The accessibility of PA opportunities in terms location, literacy and cost was a concern for some and was felt to impact some cohorts of the population more than others. For example, while the majority had no issue with paying a fee for a programme, this was a greater consideration for people on lower incomes:

*‘There are different classes around, but they are usually a bit expensive. Yoga, they’re 15 euros a class for that. So, I suppose there is a financial end to that, you know, if you’re doing one or two classes a week, the cost will add up.’ PM, 50 M*.

With regards to the location of the exercise programme, while there was a preference to exercise in less built-up areas, those in rural locations described living in areas with lower levels of walkability. Others talked about how the location needs to be part of a convenient trip chain i.e. the exercise programme would be on the way to/from another regular destination:

*‘You have to keep your wits about you. And you can’t just… you are in the countryside, you’re walking on a country road…not like you’re walking, somewhere on a greenway or something, you can gather your thoughts, you can forget about the environment’. JD, 52 M*.

##### The referrer as a safety mechanism

While participants expressed their fears of an exercise programme that is physically unsuitable or being in a group they cannot relate to, they also spoke of how a designated referrer could act as a safety mechanism and conduit to PA for those with lower self-efficacy, which could result in enhanced adherence and a reduction in vulnerability:

*‘Referring me to a person first and foremost. So that I could speak to them, and they will take into consideration my body. And then we could talk of setting out a program for me. And even if it was only a phone call, how are you progressing JC? You know, did you have any difficulties today? That they didn’t just leave you alone, and forget about you and say, “I’ll meet you in six months time”. No, because that’s too far. So, you need to connect more I suppose is the word.’ JC, 59 F*.

### Motivation

#### Reflective (beliefs) motivation

##### Integration of PA into routine habits and; competing priorities

Whilst acknowledging that motivation is inherently linked to prior areas discussed within the COM-B, participants discussed the concept of motivation broadly under the guise of addressing the need to sustain consistent PA habits. A central view held by many participants was that improvement would come about in relation to PA if PA could be integrated within their routine habits such as incidental PA. Among some participants, this was tied to the perception of a busy lifestyle, including caring and professional commitments. PA was often discussed with the inherent perception that it is a lesser priority compared to other competing priorities and therefore, not integrated into routine lifestyle behaviours:

*‘It’s hard to build it if I don’t see the purpose of it. I prefer to be active digging a hole, you know, then to go for a walk kind of thing.’ GK, 58 M*.

##### Goal setting and; self-monitoring of PA

Participants discussed facilitators to motivate engagement with PA where goal setting was a prominent determinant, whereby setting specific and achievable goals promoted a sense of achievement about PA. This could also be facilitated by self-monitoring of PA with participants describing opportunities to monitor their ascribed PA goals:

*‘I was walking for 35 minutes, and I have a Fitbit. And I was tracking my steps and training, increasing them every day.’ JD, 52 M*.

##### Feedback on PA behaviour

Participants also discussed how receiving feedback on PA performance or progress from a HCP where achievements are acknowledged alongside advice and adjustments for improvement could act as a further motivator:

*She [physiotherapist] put the exercises on the app, and it was a video of them, and then you could tick when they were done. And then the next time you saw her she could see what you’ve done. It was nice. It shows you how to do the exercise’ AJ, 44 F*.

##### Incentivisation of PA

A number of participants reported the perceived importance of *‘external reward’* in the context of motivation to improve their level of PA and a further mechanism to maintain accountability during PA. Some participants envisaged a favourable context where routine monitoring of relevant PA outcomes could allow for rewards incentivisation as part of future initiatives that seek to foster motivation. For example, some participants discussed that a future PA resource could reward PA adherence through an incentive-based structure:

*‘You kind of meet your target, could you get a voucher …Or could you get points or something that will go towards … I don’t know, like, health insurance?’ CB, 49 F*.

##### Role of self-compassion in PA engagement

Participants discussed the importance of self-compassion when engaging with PA where being kind to oneself in moments of difficulty or failure played an important role in reducing negative self-talk, building resilience and thus enhancing intrinsic motivation:

*‘There’ll be times when you’ll fall off the wagon a bit, but you just have to get back on. And, and you’re only doing this for you, you’re not doing it for somebody else, or to you know, keep a trainer happy or impress the statistics or whatever.’ AC, 54 F*.

##### Urgency for autonomy and; self-awareness in health behaviour change

There was also a noted self-awareness and sense of urgency in participants’ beliefs that their current inactive lifestyle necessitated fundamental behaviour change:

*‘Because I want to be better. Like, you know, I want to lose weight. I want to be fitter, fitter than I am right now. Because I know that will be beneficial for me.’ AC, 54 F*.

##### Intention to be physically active and; fear of morbidity and mortality

With the consideration that participants were broadly ‘inactive’ and at risk of or living with CD, there were a consistent sentiment of intention to become more physically active in the future. In most cases, this concept was tied to a desire to improve physical health status. However, some participants discussed desire for broader quality of life and mental health and wellbeing, motivated by an underlying fear of morbidity and mortality:

*‘But she* (referring to the GP) *has said you know, to do the exercise, and it will help a lot of things like mental health. But just to be more… and just physical activity, like because I was actually kind of worried about myself.’ SW, 43 F*.

*‘Because sometimes there has to be a real … well for me, the reality check of ‘If you don’t, this could be what you’re facing.’ MD, 40F*.

#### Automatic (impulses) motivation

##### Self-consciousness in PA engagement

The issue of self-consciousness was discussed by participants, where many described experiencing stigma in past experiences of engaging in PA or opportunities for PA promotion. In some cases, participants discussed where their metrics were made available to wider participants within a PA group or where interactions with HCPs involved explicit discussions about health and fitness related metrics that deviated from expected ‘healthy norms’. In other contexts, these sentiments were inherent to the individual with a physical disability engaging in PA with others, such as in a general group setting:

*‘There is also body image. Sort of the social stigma of being, you know, … with paranoia as well. You think everybody’s looking at you. So when you go for a walk, it’s like, What are they looking at? Well yeah. It’s completely crazy, like, yeah. If I was to go walking, I’d need to get walking shoes or, you know, a rain jacket or whatever it is, you know, these extras when you’re on a limited payment, when you’re on basically invalidity pension.’ AB, 53 M*.

##### Enjoyment of PA programmes

Broadly, participants held the view that PA initiatives ought to foster a sense of *‘fun’* and *‘enjoyment’* in order to support a sense of autonomous motivation for PA. Enjoyment of PA can build anticipation, driving the intrinsic psychological processes behind motivation. Having a partner to exercise with or undertaking PA in a social context were discussed as solutions for motivation for PA.

## Discussion

The research identified key determinants of PA promotion for CD in primary care from the perspective of people living with or at risk of CD. While the determinants presented are limited to the patient experience, this is an important contribution as barriers and facilitators to PA promotion in this setting have primarily been explored from the perspective of the HCP [33]. To support successful implementation, it is important to gather the perspectives of both partners in the HCP-Primary care user interaction. The findings of the present research provide valuable insights to support PA promotion efforts and inform the design of appropriate PA promotion strategies. While each determinant aligned strongly with a particular component of the COM-B, the components of the COM-B interact and influence behaviour through a dynamic interplay that influences individuals’ behaviour and opportunities for behaviour change [5]. It is also therefore important to acknowledge that the determinants highlighted may not fit solely into a single COM-B domain.

Given that experience discussed was the participants interaction with the HCP in relation to PA, many of the determinants are related to social opportunity within the COM-B model. HCPs have a unique opportunity to promote PA and motivate people at risk of or living with CD to adopt or maintain a physically active lifestyle [13]. Participants in the present research were open to their HCP raising the topic of PA. This was primarily due to the perceived credibility of HCPs and the desire for reliable information. The credibility of HCPs has been previously identified as an important dimension in the PA promotion and exercise referral process [20, 34]. This mirrors previous research in which people living with CD reported to prefer receiving PA information from their healthcare provider [35]. For primary care users, HCPs broaching the topic of PA can help to justify, motivate and facilitate behaviour change [19] [19, 20, 34] [36–41, 18, 42–44, 39].

Participants with experience of discussing PA with a HCP felt their HCP was instructing without supporting them to increase their level of PA, which likely influences the capability of individuals to understand and engage in PA effectively and thus, influences the level of motivation to initiate PA. Negative previous experiences, including where they felt judged, were disempowering and demotivating in terms of engaging in PA, a finding which is reflected in previous work, highlighting a breakdown in trust in the therapeutic alliance [45]. In contrast, a positive previous experience enhanced motivation to be physically active and provided a sense of empowerment if they felt respected by their HCP.

Similar to previous research, participants’ reported preferences for different approaches in discussing PA with HCPs reflect the individual variability in communication styles and focus of the topic (i.e. balancing fear appeals) [45]. Previous qualitative research using the COM-B model with primary care users highlighted the importance of the HCP connecting the benefits of PA to their health condition combined with the manner in which they approach the topic on the user’s acceptance of the information [19].

The expectation of tailored PA support was identified as a key determinant of the potential success or failure of PA promotion in primary care. Participants expressed the need for PA opportunities to be tailored to account for their physical ability and limitations, health conditions and individual preferences. A study by Law et al. (2021) [46] aimed to determine the mechanisms through which intervention in primary care increases PA using an evidence synthesis and co-design approach with individuals with long-term conditions, primary care professionals and relevant community professionals and researchers. Findings highlighted that individuals with long-term conditions have diverse levels of physical ability and activity, attitudes to PA and access to local resources for PA [47]. 

The findings underscore the importance of tailoring strategies to individuals to optimize engagement and motivation for PA [46], which may be facilitated by the use of Motivational Interviewing approaches within brief intervention strategies or MECC interventions [48, 49]. Indeed, participants expressed a preference for an active referral from their HCP to PA services rather than passive signposting; and wished for their HCP to receive feedback on their engagement/progress to ensure continuity of care and to give the primary care user a sense of accountabilit*y*. Similar research has reported primary care users preferences to be linked to specific opportunities rather than being advised to be more active generally [19]. Research has also highlighted that active referrals enhance adherence to PA interventions [50, 51]. Nevertheless, participants were cognisant of the capacity of the GP, which is a significant barrier to effective PA promotion [52], and were receptive to receiving information from other HCPs or support staff. People at risk or living with CD were also receptive to eHealth strategies due to the credibility of the health services and the ability to filter out misinformation. This resonates with previous studies highlighting the potential benefits of digital interventions in promoting PA among populations with CD [53, 54]. Whether the information is being relayed by the HCP or other source, the quantity and clarity of messaging is important to prevent primary care users feeling overwhelmed. This highlights the potential role of HCPs in facilitating and coordinating access to PA services as part of comprehensive care for CD prevention and management [13].

Many participants in the present research reported having no previous experience of their HCP raising the topic of PA. These findings are consistent with previous studies highlighting the limited attention given to PA in clinical encounters [36–38]. This appeared to have implications for participants psychological capability to be physically active. Participants largely viewed exercise as synonymous with weight management and therefore, felt HCPs were less inclined to raise the issue of PA if they were not overweight. This is in line with previous research that captures a weight centred focus in discussions on PA [39, 40], a bias which may lead to missed opportunities for promoting PA among individuals who may benefit, regardless of weight status. This may be linked to an apparent knowledge gap regarding exercise as medicine and the multitude of benefits of PA beyond weight loss [41]. Compounding the stigma of exercise for weight loss may also lead to lack of enjoyment and tendency to self-exclude or avoid PA and sport [43, 44], meaning that the attitudes of the HCP play a pivotal role in promoting PA in an inclusive and supportive way [39].

In relation to motivation, numerous behaviour change techniques (BCTs) were cited as mechanisms to support the adoption and maintenance of PA, including goal setting, prompts and cues, self-monitoring, feedback and incentivisation. While it may not be feasible to consider all of these within the capacity of the HCP, the HCP could potentially be supported by resources or the integration of BCTs into PA opportunities. Behaviour change techniques can assist in integrating PA into routine habits, supporting long-term maintenance. This is consistent with evidence highlighting the importance of incorporating BCTs to enhance PA engagement and maintenance [55].

A number of other features of PA opportunities were identified as determinants that may influence physical opportunities for and motivation of individuals at risk of or living with CD to be active. Accessibility was an important consideration, with regards to literacy, cost and location. When PA is accessible to participants, it enhances motivation through reducing barriers and increasing convenience and inclusivity [56]. Social support was considered a key determinant in PA behaviour change and there was a desire for the pathway to provide opportunities to engage in PA alongside people of similar ages and physical abilities and with similar health conditions. Indeed, research has demonstrated that peer modelling may support PA behaviour change through comparative thinking [57]. Moreover, social support can enhance capability through shared learning, guidance and insights offered by others, emotional support and positive reinforcement. It can also provide opportunities for encouragement, belonging, role modelling and a sense of accountability, which can enhance motivation [58].

Self-efficacy was idenitifed as a central determinant of PA promotion in this context that underpinned aspects of many other determinants. In this cohort of individuals at risk of or living with a CD, many presented with low self-efficacy to initiate or maintain PA. Enhanced self-efficacy will motivate people to engage with PA and to seek out opportunities for PA [59].

### Implications for HCPs

It is important HCPs are aware that people at risk or living with CD are amenable to them raising the topic of PA, as HCPs have previously described that their decision to instigate a PA discussion is influenced by their perception of the patient’s receptivity and openness to the topic [19]. HCPs should take a person-centred approach to support people at risk of or living with CD to initiate PA. The HCP may need to make a decision on the best approach in terms of communication style and balancing fear appeals on an individual basis and most importantly facilitate a mutually engaged discussion and positive experience. HCPs should strive to develop individuals self-efficacy by promoting PA in a positive supportive manner tailored to the individual’s health condition, physical ability and preferences. HCPs should inform people living with or at risk of CD of the benefits of PA beyond weight management, including benefits specific to their individual condition or risk of developing conditions. This may increase the value individuals place on the importance of PA. If a potential barrier to this is that HCPs themselves are lacking this knowledge [18] or the confidence in PA counselling [42]HCPs should be supported in this regard.

### Limitations

Recruitment was facilitated through the HSE communication channels potentially reaching participants that were engaged or previously engaged with health services. Therefore, this research may offer the perspectives of participants that are typically health consumers. That said, it is plausible that these are the very type of participants most likely to present in primary care and have the opportunity to engage with PA promotion. The majority of participants were highly educated, which may introduce further selection bias. Education can be a pre-disposing factor to PA promotion and those with higher education can be more likely to receive PA recommendations [60]. However, the majority did not have previous experience of receiving PA promotion from their HCP. Additionally, a number of the participants worked or were engaged with socially disadvantaged groups and provided insights into their own experiences but also from the perspective of a gatekeeper to these groups.

While participants met the eligibility criteria of 35–60 years, recruitment and data collection were performed through online platforms, which may be a barrier to older, more vulnerable and socially disadvantaged groups [54, 61]. With respect to other key demographics, the present research achieved a heterogenous balance, which is a methodological strength. Despite this, care should be taken in generalising the findings to all people with or at risk of chronic disease considering the inherent nuances associated with various conditions.

## Conclusion

This research sought to investigate the determinants of PA promotion in primary care from the perspective of individuals at risk of and living with CD. Guided by the COM-B, the findings demonstrate the complex interplay between Capability, Opportunity and Motivation in relation to PA promotion for CD, where elements of each construct operate with a backdrop of intervening and shifting variables. Indeed, the results highlight that while motivation is critical for this cohort, this cannot be facilitated without building capability, which is preceded by the creation of opportunity through supportive environments. The COM-B model provided a valuable framework for capturing key determinants and representing the views and insights gained from participants. The analysis emphasises the value of HCPs being supported to broach the issue of PA in a therapeutic and person-centred manner using diverse and flexible approaches. The analysis also highlights the importance of accessible, PA opportunities, focused on enhancing self-efficacy and fostering social support through behaviour change techniques to assist in addressing the diverse needs and preferences of individuals in terms of their PA journey. The findings from the current research provide learnings that can support PA promotion efforts and the development of future PA promotion strategies or resources for primary health care to support those at risk of or living with CD to maintain or become physically active.

### Electronic supplementary material

Below is the link to the electronic supplementary material.

Supplementary Material 1

Supplementary Material 2

## Data Availability

The dataset supporting the conclusions of this article are available from the corresponding author upon reasonable request.

## Abbreviations

BCTs: Behaviour change techniques
BCW: Behaviour Change Wheel
CD: Chronic disease
GP: General practitioner
HCP: Healthcare professional
HSE: Health Service Executive
MECC: Make Every Contact Count
PA: Physical activity
WHO: World Health Organisation
