# Supplementary material for: Determinants of physical activity promotion in primary care from the patient perspective of people at risk of or living with chronic disease: a COM-B analysis
Source: BMC Prim Care. 2024 May 28;25:190. doi: 10.1186/s12875-024-02440-2 (PMC11134685; doi:10.1186/s12875-024-02440-2)
Supplement: Supplementary file 1 — Supplementary Material 1 [file 12875_2024_2440_MOESM1_ESM.docx]

**Supplementary File 1: Interview Schedule**

Define healthcare provider (HCP) as any HCP they come in contact with in the community, such as a GP, practice nurse, occupational therapist or dietician, etc.

Define physical activity (PA) was defined as interchangeable with exercise and examples of PA were provided (e.g. walking for transport or recreation, gardening, cycling, yoga, swimming, jogging and running).

| **Concept/Objective** | **Questions, Topics and Probes** | **COM-B dimension** |
| --- | --- | --- |
| Introduction | **In the screening questionnaire, you said that you usually do at least 30 minutes of exercise on X days a week. How do you feel about the amount of exercise you do at the minute?**   - What are some of the reasons or things that stop you from doing more?   *Probes; Physical limitations, motivation, social support, resources, knowledge*   - What would make it easier for you to do more exercise? | Capability  Opportunity  Motivation |
| Establish how recently they interacted with healthcare provider | **I’m interested in hearing about your experiences of interacting with HCPs. Can I firstly ask you what HCPs you have attended over the last year?**  *Probes; GP and/or PN? If no, others? Physio, Dietician, OT, time in outpatient dept* |  |
| What do people at risk of or living with CD **experience when discussing their physical activity** *(or other behaviours)* with a healthcare provider? | **Has your GP or PN (or any of the other HCPs) ever discussed a health behaviour with you? Maybe something like exercise, what you eat, smoking, drinking? What was your experience of that discussion?** *[Focus conversation on exercise if they have experience of it. If not choose another behaviour*   - If no, how would you feel if they did raise the topic of your health behaviours with you? - If yes, who with? - How was the conversation brought up? What did they say? How did you feel during and after the chat about exercise *(or other behaviour)*? Why? - How appropriate did you feel it was for this healthcare provider to discuss the topic of exercise *(or other behaviour)* with you?   *Probes; Who should bring it up (repeat list), when should they bring it up*   - What could they have done differently?   *Probes; guidance, understanding, advice, empathy, support*   - Did the conversation prompt you to start exercise, explore exercise options, change any behaviours? | Capability  Opportunity |
|  | **What’s the best way to bring up the topic of exercise? What do you think of these 2 ways? Which of these approaches would you respond to best?**   1. Would it be ok if we spend a couple of minutes talking about exercise and how it might help your health? What do you already know about how exercise might help? 2. I think that exercise could really help your health. I’m going to explain some of the ways that it might do that.   **What do you make of both of those?** *[Repeat and probe – why]* | Capability  Motivation |
| What do people at risk or and/or living with CD **want to happen when discussing physical activity** with a healthcare provider? | **If more healthcare providers in Ireland started bringing up the topic of exercise with patients, what would you expect to happen in the conversation?**   - Would you prefer to be told information or to be asked? - What would you like to be discussed?   *Probes; Benefits of exercise, risks of not exercising, how to start exercising, an exercise programme, a referral to someone else that would have more time to talk to you about exercise, a referral to an exercise programme or facility near where you live. – Probe most important and why]*   - Sometimes people say that when a [healthcare provider] tells them about the risks of not exercising and stresses the importance of exercise to them, it can really motivate them to make a change. What do you think? - How long would you expect the conversation to be? - How confident would you be that your healthcare provider would have the expertise to have a detailed discussion about this? Which ones? - What would you like your healthcare professional to know about your physical activity preferences or goals? [record of PA history, continuing discussion at follow up visit] - That’s a lot of things your HCP could do – asking you about your exercise, advising you exercise more, checking how ready you are to exercise and helping you decide what exercise to do. Which of these tasks should be done by the HCP? | Motivation  Opportunity |
| What do people at risk or and/or living with CD **want to happen when receiving a physical activity** referral from a healthcare provider? | **Let's chat more about what being referred somewhere might look like.**  **Have you ever been signposted or referred to a physical activity program or resource by a healthcare professional?** *(If yes)* **tell me about that.** *(If no)* have you signposted or referred for other services by a healthcare provider? How did that work? (how was the referral handled?)  **What would be the best WAY to refer you to physical activity services such as a fitness instructor, physical activity group or online information ?**  *Use photopeak diagram to explain 6 possible* ***ways*** *of referring someone*   - Which of these would be best do you think? Why? - Formal Prescribing Referral - Would it be ok if that referral was done electronically i.e. the GP or PN was able to refer you somewhere by clicking a button on their PC *[essential to ask/probe this]* - How important is it for the HCP to explain the reason the referral is being made?   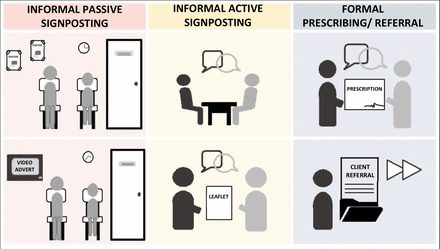 |  |
| The design of a referral process | **There are so many ways to help people exercise more. I might mention some of the more popular ones and then you can tell me which ones would interest you. So you could be referred or signposted to;**  a fitness instructor or personal trainer in a nearby gym, to a group that meet regularly for activities like walking, swimming or some other type of sport, it could be to a sports club, it could just learning about places for you to be active in your local community, like walking trails and parks, gyms, swimming pools. Some people even just like having ideas to increase their step count without doing any real exercise.  **What do you make of those ideas? If you ever did decide to exercise more, what might work for you?**  *Probe; individual v group, indoor v outdoors, facility v non-facility, wearables,*  *How important is the cost (e.g. of equipment, classes, specialised gear) to you to influence your decision to exercise or affect your motivation to continue exercising?*   - How do you feel about support with directing you to exercise options?   *Probe; How important is it that you speak to someone about exercise options?*   - What else could help motivate you to exercise?   *Probes; Like helping you to set and track exercise goals, having a way to chat to an exercise professional, keeping a diary of your progress, getting feedback on changes in your health, building rewards into your programme; fitness apps and/or online exercise videos*   - Would the exercise options need to be specifically for patients with chronic disease?   *Probe; why and how*   - After you are referred somewhere or the programme you were referred to ends, what happens then?   *Probe; should you be signposted to new options? How important is it that your HCP be kept up to date with your progress?* | Opportunity  Motivation |
| **Closing** | **Is there anything else you feel that we should have discussed but didn’t?** |  |

Note: CD, chronic disease; GP, general practitioner; HCP, healthcare provider; OT, occupational therapist; physio, physiotherapist; PN, practice nurse
