# Supplementary material for: Determinants of physical activity promotion in primary care from the patient perspective of people at risk of or living with chronic disease: a COM-B analysis
Source: BMC Prim Care. 2024 May 28;25:190. doi: 10.1186/s12875-024-02440-2 (PMC11134685; doi:10.1186/s12875-024-02440-2)
Supplement: Supplementary file 2 — Supplementary Material 2 [file 12875_2024_2440_MOESM2_ESM.docx]

## Supplementary File 2: Participant Demographics

Table: Participant demographics (n=22)

| Age (years) | 49.18±5.83 |  |
| --- | --- | --- |
| Gender |  |  |
| Male | 11(50%) |  |
| Female | 11(50%) |  |
| Residential setting |  |  |
| Urban | 12(55%) |  |
| Rural | 10(45%) |  |
| Educational status |  |  |
| NFQ Level 4: Upper secondary education | 1(4%) |  |
| NFQ Level 6: Higher certificate | 3(14%) |  |
| NFQ Level 7: Ordinary bachelor degree/national diploma | 3(14%) |  |
| NFQ Level 8: Honours bachelor degree/professional qualification | 6(27%) |  |
| NFQ Level 9: Postgraduate diploma, master degree or equivalent | 6(27%) |  |
| NFQ Level 10: Doctorate (Ph.D) or higher | 3(14%) |  |
| Health status |  |  |
| Obesity | 7(32%) |  |
| Arthritis | 3(14%) |  |
| Diabetes | 2(9%) |  |
| Cardiovascular disease | 1(4%) |  |
| Muscular dystrophy | 1(4%) |  |
| Cancer | 1(4%) |  |
| Liver transplant recipient | 1(4%) |  |
| No chronic disease | 6(27%) |  |
| Physical activity status |  |  |
| Meeting the physical activity guidelines | 0(0%) |  |
| Not meeting the physical activity guidelines | 22(100%) |  |

Continuous variables displayed as mean±standard deviation. Categorical variables displayed as frequency (percentage).

NFQ; national framework of qualifications
